# Medication literacy and its associated factors among middle-aged and older patients with comorbid coronary heart disease and type 2 diabetes mellitus: a cross-sectional study

**DOI:** 10.3389/fpubh.2026.1851844

**Published:** 2026-09-23

**Authors:** Xiao Pan, Xili Zhao, Yingqi Dai, Jiaxin Wei

**Affiliations:** 1 The Second Affiliated Hospital of Chongqing Medical University, Chongqing, China; 2 Chongqing Geriatric Specialized Nursing Clinical Diagnosis and Treatment Center, Chongqing, China; 3 Acute and Critical Care Nursing Innovation and Translation Engineering Research Center of Chongqing Education Commission of China, Chongqing, China

**Keywords:** associated factors, coronary heart disease, cross-sectional study, medication literacy, type 2 diabetes mellitus

## Abstract

**Objective:**

To assess medication literacy among middle-aged and older patients with comorbid coronary heart disease (CHD) and type 2 diabetes mellitus (T2DM).

**Methods:**

A cross-sectional survey was conducted using convenience sampling. From October 2024 to May 2025, 260 hospitalized middle-aged and older patients with comorbid CHD and T2DM were recruited from a tertiary Grade A hospital in Chongqing, China. Data were collected using a general information questionnaire, the Medication Literacy Self-Assessment Scale for Patients with Comorbid Coronary Heart Disease and Diabetes, the Social Support Rating Scale, the Self-Efficacy for Appropriate Medication Use Scale, and the Patient Participation in Medication Decision-Making Scale. Descriptive statistics, univariate analysis, Pearson correlation analysis, and hierarchical linear regression analysis were performed.

**Results:**

A total of 254 valid questionnaires were included in the final analysis. The mean medication literacy score was 74.05 ± 12.23. Hierarchical linear regression analysis showed that participation in medication decision-making, social support level, educational level, self-efficacy for appropriate medication use, place of residence, and the ability to understand drug instructions were significantly associated factors of medication literacy (all *p* < 0.05).

**Conclusion:**

Medication literacy among middle-aged and older patients with comorbid CHD and T2DM has room for further improvement. It was associated with factors across individual, social, cognitive and functional, and healthcare system levels. Individualized interventions and multilevel support systems should be developed to improve medication literacy and promote medication safety in this population.

## 1 Introduction

Against the backdrop of the growing prevalence of chronic non-communicable diseases and accelerated population aging, multimorbidity among middle-aged and older adults has become a major challenge in clinical practice and public health management. Coronary heart disease (CHD) and type 2 diabetes mellitus (T2DM) are not only two of the most common chronic diseases, but also frequently coexist and impose a substantial disease burden (1, 2). Through mechanisms such as dysregulated glucose and lipid metabolism and endothelial dysfunction, these two conditions may interact and aggravate each other, resulting in a markedly increased risk of cardiovascular events, more severe target-organ damage, and poorer clinical outcomes (3). Previous studies have shown that 37.6% of hospitalized patients with acute coronary syndrome had concomitant diabetes or abnormal glucose metabolism, which was associated with a 1.5-fold increase in the risk of major adverse cardiovascular events and a twofold increase in all-cause mortality (3). Therefore, the burden associated with this comorbidity is greater than that of either disease alone and places higher demands on clinical treatment and long-term management.

Medication therapy is a core component of disease management in middle-aged and older patients with comorbid CHD and T2DM. Compared with patients with either CHD or T2DM alone, those with both conditions often require long-term combination regimens that include antiplatelet agents, lipid-lowering and plaque-stabilizing medications, glucose-lowering drugs, and anti-ischemic therapies (4). This leads to more complex treatment plans, a higher risk of drug interactions and adverse drug reactions, and greater challenges in medication self-management (5). Accordingly, comorbid status places higher demands on patients’ ability to comprehend medication information, weigh treatment benefits against risks, and manage adverse reactions. After discharge, these patients must handle most medication tasks independently at home. Evidence shows that approximately 16% of hospital readmissions stem from medication-related issues, 40% of which are preventable through effective interventions (6). In addition, up to 50% of older adults experience at least one medication error within 90 days after discharge (7). If middle-aged and older patients with comorbid CHD and T2DM lack sufficient ability to access, understand, and apply medication-related information in such a complex comorbidity context, they are more likely to missed doses, incorrect administration, unauthorized discontinuation, arbitrary dose adjustment, and show poor adherence (8). These problems may compromise disease control, increase the risk of medication-related adverse events, and result in unnecessary healthcare resource utilization (9).

Medication literacy is an extension of health literacy in the field of pharmacotherapy. It is defined as an individual’s comprehensive ability to obtain, understand, communicate, calculate, and process medication-related information to make appropriate medication decisions and behaviors (10). Studies have confirmed that good medication literacy is closely associated with higher medication adherence, more positive beliefs about medicines, and stronger medication self-management ability (11–13). Additionally, medication literacy has been found to be positively correlated with quality of life in patients with chronic multimorbidity (14). Therefore, medication literacy is not only closely associated with ensured medication safety and improving disease management outcomes, but also a key entry point for prevention and control of chronic diseases and targeted health education interventions.

However, existing research on medication literacy mostly focuses on patients with a single chronic disease, or treats patients with multiple chronic conditions as a relatively homogeneous group. In-depth investigations of specific comorbidity combinations remain scarce. Furthermore, previous studies often use universal measurement tools lacking disease specificity, and their analysis of associated factors is frequently limited to one or a few dimensions. The conceptual model of medication literacy posits associations between medication literacy and four categories of antecedents, namely individual, social, cognitive-functional, and healthcare system-related factors (15). Within this framework, these four sets of factors are hypothesized to correlate with the core and subdimensions of medication literacy, and further link to patients’ processing of medication-related information, which may correspond to safer and more appropriate medication use. This integrated framework provides a comprehensive perspective for analyzing the factors associated with medication literacy in patients with comorbid CHD and T2DM. Accordingly, this study investigated the current status of medication literacy among middle-aged and older patients with comorbid CHD and T2DM, and analyzed its associated factors based on the four domains of the medication literacy conceptual model. The findings aim to provide reference for developing targeted and systematic intervention strategies to improve medication literacy.

## 2 Materials and methods

### 2.1 Study design and participants

This cross-sectional study was conducted using a convenience sampling approach. Study participants were recruited from inpatients diagnosed with both coronary heart disease and type 2 diabetes at a tertiary Grade A hospital in Chongqing from October 2024 to May 2025.

Inclusion criteria for participants were as follows: (1) age ≥ 45 years; (2) met the American Heart Association/American College of Cardiology diagnostic criteria for CHD (16), and concurrently met the diagnostic criteria for T2DM specified in the *2020 Chinese Guidelines for the Prevention and Treatment of Type 2 Diabetes Mellitus* (17); (3) had taken medications for CHD and T2DM for ≥ 3 months; (4) participating voluntarily. The exclusion criteria were as follows: (1) had severe cognitive, hearing, or speech impairment, or severe psychiatric disorders that prevented them from completing the survey; (2) had life-threatening diseases; (3) were participating in other drug-related studies.

Based on Kendall’s sample size principle (18), the sample size for determinant analysis should be 5–10 times the number of variables. This study considered 24 variables, and after factoring in a 20% rate of invalid questionnaires, a minimum of 150 participants was deemed necessary. The study protocol was approved by the Ethics Committee of the Second Affiliated Hospital of Chongqing Medical University (Approval No. 2025–119).

### 2.2 Measures

#### 2.2.1 General information questionnaire

It covered sociodemographic characteristics including gender, age, educational level, employment status, place of residence, living arrangement, monthly per capita household income, marital status, and medical payment method, as well as clinical characteristics.

#### 2.2.2 Medication literacy self-assessment scale for patients with comorbid coronary heart disease and diabetes

This scale was developed by Liu et al. (19) in 2024 to assess medication literacy among adult patients with comorbid CHD and diabetes. It contains 23 items across five dimensions: access, understand, evaluate, calculate, and communicate. It employs a 5-point Likert scale, with higher scores indicating better medication literacy. The original study reported an overall Cronbach’s *α* coefficient is 0.911, test–retest reliability of 0.948, and a content validity index of 0.997. Confirmatory factor analysis confirmed good model fit. In the present study, the Cronbach’s *α* coefficient for this scale was 0.940.

#### 2.2.3 Social support rating scale

This scale was developed by Xiao (20) based on international social support scales and is used to assess patients’ social support status. It comprises 10 items distributed across three domains: objective support (3 items), subjective support (4 items), and support utilization (3 items). The overall score is calculated by summing responses to all items, with scores of 22 or below indicating low social support, 23–44 indicating a moderate level, and 45–66 indicating a high level. Higher total scores reflect greater social support. In the present study, the Cronbach’s *α* coefficient of the scale was 0.732.

#### 2.2.4 Self-efficacy for appropriate medication use scale

This scale was originally developed by Risser et al. (21)in 2007 and introduced into China by Dong et al. (22) in 2015. It is primarily used to assess patients’ self-efficacy regarding rational medication use. The scale contains 13 items rated on a 3-point Likert scale. The total score ranges from 13 to 39, with higher scores indicating greater rational medication self-efficacy. In this study, the Cronbach’s *α* coefficient was 0.733.

#### 2.2.5 Patient participation in medication decision-making scale

This scale was developed by Wang et al. (23) based on the 9-item Shared Decision-Making Questionnaire (SDM-Q-9) adapted by Kriston et al. (24). It is used to assess the extent of patient participation in medication treatment decisions. The revised scale consists of 9 items rated on a 5-point Likert scale. Response options range from “strongly agree” to “strongly disagree” scored from 1 to 5, respectively. The total score ranges from 9 to 45, with higher scores indicating lower levels of participation in medication decision-making. The original Cronbach’s *α* coefficient was 0.847. In this study, we reverse-scored all items of this scale to ensure consistent score interpretation across all scales used. Specifically, we converted the original response values (1–5) to reverse scores (5–1), so that higher total scores now reflect greater patient participation. In the present sample, Cronbach’s *α* coefficient for this scale was 0.857.

### 2.3 Data collection

After obtaining permission from the heads of the relevant hospital departments, trained members of the research team collected data through face-to-face questionnaire surveys. Before data collection, participants received a standardized explanation of the study purpose, survey procedures, and key points for completing the questionnaire. After written informed consent was obtained, anonymous paper questionnaires were distributed for self-completion. For patients who had difficulty completing the questionnaire, the researchers read each item aloud verbatim and recorded responses truthfully according to the patients’ answers. After collection, the completeness of each questionnaire was checked on-site, and any missing or unclear items were promptly clarified with the patient to supplement necessary information. Data were double-entered and verified by two researchers, and questionnaires with logical errors or patterned responses were excluded.

### 2.4 Statistical analysis

All data were entered into Excel 2021 and analyzed using SPSS 27.0. Categorical variables were described using frequencies and percentages, and normally distributed continuous variables were summarized as mean ± standard deviation. Comparisons between groups were performed using the independent-samples t-test or one-way analysis of variance; and non-parametric tests for non-normal data. Pearson correlation was used to analyze associations between medication literacy and social support, self-efficacy for appropriate medication, and degree of participation in medication decision-making. Hierarchical linear regression analysis was then used to determine the independent factors associated with medication literacy. Statistical significance was defined as *p* < 0.05.

## 3 Results

### 3.1 Participant characteristics

A total of 260 questionnaires were distributed, and six were excluded (three due to patient withdrawal and three due to patterned responses). The remaining 254 valid questionnaires were included in the final analysis, yielding an effective response rate of 97.69%. Patients ranged in age from 45 to 85 years, with a mean age of 67.66 ± 8.29 years. Table 1 presents comprehensive sociodemographic and disease-related characteristics.

**Table 1:** Univariate analysis of associated factors of medication literacy (*n* = 254).

Variable	Group	*n*	%	medication literacy score (mean + SD)	*t/F*	*p*
Gender	Male	136	53.54	75.06 ± 11.95	1.419	0.157
	Female	118	46.46	72.88 ± 12.48		
Age (years)	45 to 59	47	18.50	82.06 ± 12.80	17.035	<0.001
	60 to 69	89	35.04	75.64 ± 11.60		
	70 to 79	94	37.01	71.04 ± 1 0.42		
	≥80	24	9.45	64.21 ± 9.15		
Educational level	Primary school or below	59	23.23	62.44 ± 7.91	66.808	<0.001
	Junior high school	98	38.58	72.04 ± 9.72		
	Senior high school/technical secondary school	57	22.44	81.28 ± 9.37		
	College or above	40	15.75	85.78 ± 9.18		
Employment status	Employed	41	16.14	86.20 ± 11.05	7.708	<0.001
	Unemployed/Retired	213	83.86	71.71 ± 11.01		
Place of residence	Urban	205	80.71	76.80 ± 11.63	8.238	<0.001
	Rural	49	19.29	62.55 ± 6.76		
Living arrangement	Living with spouse	153	60.24	75.54 ± 12.28	2.924	0.035
	Living with children	46	18.11	69.78 ± 10.68		
	Living alone	28	11.02	74.71 ± 12.27		
	Other	27	10.63	72.19 ± 13.04		
Monthly per capita household income (CNY)	<3,000	83	32.68	66.93 ± 9.32	45.397	<0.001
	3,000 to 5,000	99	38.98	73.48 ± 10.76		
	>5,000	72	28.35	83.03 ± 11.44		
Marital status	Married	186	73.23	74.77 ± 12.40	1.560	0.120
	Divorced/widowed/unmarried	68	26.77	72.07 ± 11.58		
Medical payment method	Urban employee basic medical insurance	168	66.14	78.10 ± 11.62	8.958	<0.001
	Urban–rural resident basic medical insurance/Other	86	33.86	66.14 ± 9.172		
Family history	Yes	49	19.29	77.02 ± 11.68	1.905	0.058
	No	205	80.71	73.34 ± 12.27		
Disease duration (years)	≤5	138	54.33	74.86 ± 12.67	5.504	0.005
	6 to 10	75	29.53	75.19 ± 12.16		
	>10	41	16.14	69.22 ± 9.66		
Years of medication use	≤5	58	22.83	78.52 ± 12.77	6.574	0.002
	6 to 10	103	40.55	74.02 ± 12.73		
	>10	93	36.61	71.29 ± 10.49		
Number of other comorbid chronic diseases	0	39	15.35	78.72 ± 12.80	9.609	<0.001
	1 to 2	167	65.75	74.70 ± 11.81		
	≥3	48	18.90	67.98 ± 11.04		
Number of medications taken daily	≤5	120	47.24	75.68 ± 12.81	2.031	0.043
	>5	134	52.76	72.58 ± 11.53		
Frequency of daily medication use	1 time/day	49	19.29	75.45 ± 11.77	0.507	0.603
	2 times/day	97	38.19	74.13 ± 13.17		
	≥3 times/day	108	42.52	73.33 ± 11.59		
Number of hospitalizations due to CHD and T2DM	≤4	126	49.61	74.52 ± 12.92	1.080	0.341
	5 to 8	93	36.61	74.47 ± 11.53		
	≥9	35	13.78	71.23 ± 11.36		
History of adverse drug reactions to CHD or T2DM medications	Yes	68	26.77	76.34 ± 12.66	1.814	0.071
	No	186	73.23	73.21 ± 11.99		
Self-perceived disease control	Poor	58	22.83	69.97 ± 10.32	5.008	0.007
	Fair	104	40.94	74.31 ± 12.81		
	Good	92	36.22	76.33 ± 12.13		
Level of detail of medication education received	Not detailed	63	24.80	68.27 ± 10.81	17.553	<0.001
	Relatively detailed	113	44.49	73.37 ± 11.04		
	Very detailed	78	30.71	79.69 ± 12.63		
Frequency of reading drug instructions	Rarely	70	27.56	66.36 ± 10.55	37.344	<0.001
	Occasionally	103	40.55	73.42 ± 11.31		
	Frequently	81	31.89	81.49 ± 10.26		
Ability to understand drug instructions	Cannot understand / unclear	47	18.50	63.30 ± 8.81	68.458	<0.001
	Partially understand	140	55.12	72.56 ± 10.53		
	Fully understand	67	26.38	84.70 ± 9.11		

### 3.2 The current status of medication literacy

Among the 254 middle-aged and older patients with CHD and T2DM, the total medication literacy score was 74.05 ± 12.23, with a mean item score of 3.22 ± 0.53. Across the five dimensions, the mean item scores ranked from highest to lowest as follows: calculate (3.39 ± 0.55), understand (3.34 ± 0.62), evaluate (3.24 ± 0.62), communicate (3.05 ± 0.61), and access (2.96 ± 0.72) (Table 2).

**Table 2:** The current status of medication literacy (*n* = 254).

Variable	Number of items	Possible score range	Observed score range	Mean total score	Mean item score
Medication literacy	23	23 to 115	50 to 99	74.05 ± 12.23	3.22 ± 0.53
Access	5	5 to 25	7 to 23	14.81 ± 4.32	2.96 ± 0.72
Understand	7	7 to 35	14 to 32	23.40 ± 4.35	3.34 ± 0.62
Communicate	3	3 to 15	4 to 14	9.15 ± 1.83	3.05 ± 0.61
Evaluate	3	3 to 15	5 to 14	9.72 ± 1.85	3.24 ± 0.62
Calculate	5	5 to 25	9 to 24	16.93 ± 2.60	3.39 ± 0.55

### 3.3 Univariate analysis of associated factors of medication literacy

Univariate analysis showed that medication literacy scores differed significantly according to age, educational level, employment status, place of residence, monthly per capita household income, medical payment method, disease duration, years of medication use, number of other comorbid chronic diseases, number of medications taken daily, self-perceived disease control, level of detail of medication education received, frequency of reading drug instructions, and ability to understand drug instructions (all *p* < 0.05; Table 1).

### 3.4 Correlation between variables

The mean social support score was 35.82 ± 6.07, the mean self‑efficacy for appropriate medication use scale score was 26.88 ± 3.40, and the mean score for patient participation in medication decision-making was 25.76 ± 5.78. Pearson correlation analysis showed that medication literacy was positively correlated with social support, self-efficacy for appropriate medication use, and the level of patient participation in medication decision-making (r = 0.595, 0.690, 0.753, all *p* < 0.01; Table 3).

**Table 3:** Correlation between variables (*n* = 254).

Variable	1	2	3	4
1. Medication literacy	1	0.595**	0.690**	0.753**
2. Social support	0.595**	1	0.516**	0.461**
3. Self-efficacy for appropriate medication use	0.690**	0.516**	1	0.630**
4. Patient participation in medication decision-making	0.753**	0.461**	0.630**	1

**p < 0.01.

### 3.5 Hierarchical linear regression analysis of factors associated with medication literacy

In this study, medication literacy score was used as the dependent variable. Variables with statistical significance in both univariate analysis and Pearson correlation analysis (all *p* < 0.05) were included as independent variables. These variables were assigned to four hierarchical blocks and entered sequentially into the multiple linear regression model: individual factors as the first block of independent variables, social factors as the second block, cognitive and functional factors as the third block, and health system factors as the fourth block. The method of assigning independent variables is shown in Table 4. The results showed that educational level, social support, place of residence, self-efficacy for appropriate medication use, ability to understand drug instructions, and degree of patient participation in medication decision-making were significantly associated with medication literacy (all *p* < 0.05). The final adjusted model explained 75.0% of the variance in medication literacy among middle-aged and older patients with comorbid CHD and T2DM. Harman’s one-way test showed that there were 7 factors with eigenvalues greater than 1, and the first factor only explained 29.53% of the variance, indicating that there was no significant common method bias in this study. Multicollinearity diagnostics showed that the variance inflation factor (VIF) for all retained variables was < 10 and the tolerance was > 0.1, indicating no serious multicollinearity. The Durbin-Watson statistic was1.889, which was close to 2, suggesting no obvious autocorrelation in the residuals. Residual analysis showed that the standardized residual histogram was approximately normally distributed, and the points in the normal *P–P* plot were distributed close to the diagonal line, indicating satisfactory normality of residuals. In the scatterplot of standardized residuals versus standardized predicted values, the points were randomly distributed around zero, indicating homoscedasticity. Cook’s distance ranged from 0.000 to 0.050, all < 1. Therefore, the basic assumptions of multiple linear regression were met (Table 5).

**Table 4:** Assignment methods of independent variables.

Variable	Description of the assignment
Age (years)	45 to 59 = 1, 60 to 69 = 2, 70 to 79 = 3, ≥80 = 4
Educational level	Primary school or below = 1, Junior high school = 2, Senior high school/technical secondary school = 3, College or above = 4
Employment status	Employed = 0, Unemployed/Retired = 1
Place of residence	Urban = 0, Rural = 1
Living arrangement	Living with spouse (0, 0, 0, 0), Living with children (0, 1, 0, 0), Living alone (0, 0, 1, 0), Other (0, 0, 0, 1)
Monthly per capita household income(CNY)	<3,000 = 1, 3,000 to 5,000 = 2, >5,000 = 3
Medical payment method	Urban employee basic medical insurance = 0, Urban–rural resident basic medical insurance/Other = 1
Disease duration (years)	≤5 = 1, 6 to 10 = 2, >10 = 3
Years of medication use	≤5 = 1, 6 to 10 = 2, >10 = 3
Number of other comorbid chronic diseases	0 = 1, 1 to 2 = 2, ≥3 = 3
Number of medications taken daily	≤5 = 1, >5 = 2
Self-perceived disease control	Poor = 1, Fair = 2, Good = 3
Level of detail of medication education received	Not detailed = 1, Relatively detailed = 2, Very detailed = 3
Frequency of reading drug instructions	Rarely = 1, Occasionally = 2, Frequently = 3
Ability to understand drug instructions	Cannot understand/unclear = 1, Partially understand = 2, Fully understand = 3
Social support	Input as original value
Self-efficacy for appropriate medication use	Input as original value
Patient participation in medication decision-making	Input as original value

**Table 5:** Results of the hierarchical linear regression analysis on variables associated with medication literacy (*n* = 254).

Model	Variables	*B* (95% CI)	*SE*	*β*	*t*	*p*	*R* ^2^	Adjusted *R*^2^	Δ*R*^2^	*F*	*p*
Model 1	(Constant)	63.146 (52.955, 73.337)	5.174	—	12.205	<0.001	0.526	0.509	0.526	30.143	<0.001
	Age (years)	−1.935 (−3.849, −0.021)	0.972	−0.141	−1.991	0.048					
	Education level	6.231 (4.937, 7.524)	0.657	0.509	9.486	<0.001					
	Unemployed/ Retired	−3.793 (−7.474, −0.111)	1.869	−0.114	−2.029	0.044					
	Monthly per capita household income(CNY)	1.962 (0.195, 3.729)	0.897	0.125	2.187	0.030					
	Disease duration(years)	0.627 (−1.533, 2.786)	1.096	0.038	0.572	0.568					
	Years of medication use	0.069 (−1.859, 1.997)	0.979	0.004	0.071	0.944					
	Number of other comorbid chronic diseases	−1.084 (−3.570, 1.401)	1.262	−0.052	−0.860	0.391					
	Number of medications taken daily	−0.290 (−4.104, 3.524)	1.936	−0.007	−0.150	0.881					
	Self-perceived disease control	0.964 (−0.545, 2.474)	0.766	0.060	1.258	0.209					
Model 2	(Constant)	40.445 (27.140, 53.751)	6.754	—	5.988	<0.001	0.639	0.618	0.113	30.236	<0.001
	Age (years)	−1.392 (−3.242, 0.458)	0.939	−0.102	−1.482	0.140					
	Education level	4.631 (3.393, 5.869)	0.628	0.378	7.369	<0.001					
	Unemployed/ Retired	−0.440 (−3.859, 2.978)	1.735	−0.013	−0.254	0.800					
	Monthly per capita household income(CNY)	0.782 (−0.814, 2.379)	0.811	0.050	0.965	0.335					
	Disease duration (years)	−0.584 (−2.543, 1.376)	0.995	−0.036	−0.587	0.558					
	Years of medication use	0.337 (−1.376, 2.050)	0.869	0.021	0.388	0.699					
	Number of other comorbid chronic diseases	−0.214 (−2.428, 2.000)	1.124	−0.010	−0.190	0.849					
	Number of medications taken daily	−0.065 (−3.520, 3.391)	1.754	−0.002	−0.037	0.971					
	Self-perceived disease control	0.892 (−0.463, 2.247)	0.688	0.055	1.297	0.196					
	Place of residence	−7.063 (−9.926, −4.199)	1.454	−0.228	−4.859	<0.001					
	Living with children	2.267 (−0.785, 5.319)	1.549	0.072	1.463	0.145					
	Living alone	3.050 (−0.244, 6.345)	1.672	0.078	1.824	0.069					
	Other	−2.616 (−5.907, 0.674)	1.670	−0.066	−1.566	0.119					
	Social support	0.693 (0.475, 0.912)	0.111	0.344	6.246	<0.001					
Model 3	(Constant)	13.117 (−0.930, 27.163)	7.130	—	1.840	0.067	0.711	0.691	0.072	34.208	<0.001
	Age (years)	0.551 (−1.243, 2.344)	0.911	0.040	0.605	0.546					
	Education level	3.140 (1.794, 4.486)	0.683	0.256	4.596	<0.001					
	Unemployed/ Retired	−1.481 (−4.607, 1.644)	1.587	−0.045	−0.934	0.351					
	Monthly per capita household income(CNY)	0.197 (−1.280, 1.674)	0.750	0.013	0.262	0.793					
	Disease duration (years)	−1.081 (−2.866, 0.704)	0.906	−0.066	−1.193	0.234					
	Years of medication use	−0.165 (−1.719, 1.389)	0.789	−0.010	−0.210	0.834					
	Number of other comorbid chronic diseases	0.069 (−1.927, 2.064)	1.013	0.003	0.068	0.946					
	Number of medications taken daily	1.093 (−2.064, 4.250)	1.602	0.027	0.682	0.496					
	Self-perceived disease control	0.667 (−0.555, 1.888)	0.620	0.041	1.076	0.283					
	Place of residence	−5.663 (−8.280, −3.046)	1.328	−0.183	−4.263	<0.001					
	Living with children	0.745 (−2.045, 3.536)	1.416	0.024	0.526	0.599					
	Living alone	0.696 (−2.333, 3.726)	1.538	0.018	0.453	0.651					
	Other	−1.690 (−4.669, 1.289)	1.512	−0.043	−1.118	0.265					
	Social support	0.471 (0.264, 0.677)	0.105	0.234	4.491	<0.001					
	Frequency of reading drug instructions	−0.368 (−1.872, 1.137)	0.764	−0.023	−0.481	0.631					
	Ability to understand drug instructions	2.443 (0.553, 4.333)	0.959	0.133	2.547	0.012					
	Self-efficacy for appropriate medication use	1.174 (0.840, 1.508)	0.170	0.327	6.928	<0.001					
Model 4	(Constant)	19.311 (6.435, 32.187)	6.535	—	2.955	0.003	0.770	0.750	0.058	38.955	<0.001
	Age (years)	−0.098 (−1.721, 1.526)	0.824	−0.007	−0.118	0.906					
	Education level	2.676 (1.450, 3.902)	0.622	0.218	4.301	<0.001					
	Unemployed/ Retired	−1.325 (−4.139, 1.490)	1.429	−0.040	−0.927	0.355					
	Monthly per capita household income(CNY)	−0.374 (−1.740, 0.992)	0.693	−0.024	−0.539	0.590					
	Disease duration (years)	−0.720 (−2.351, 0.911)	0.828	−0.044	−0.870	0.385					
	Years of medication use	0.140 (−1.271, 1.551)	0.716	0.009	0.195	0.845					
	Number of other comorbid chronic diseases	−0.383 (−2.184, 1.419)	0.914	−0.018	−0.418	0.676					
	Number of medications taken daily	−0.202 (−3.060, 2.656)	1.451	−0.005	−0.139	0.889					
	Self-perceived disease control	0.737 (−0.362, 1.836)	0.557	0.046	1.321	0.188					
	place of residence	−3.886 (−6.332, −1.440)	1.242	−0.126	−3.130	0.002					
	Living with children	1.306 (−1.207, 3.819)	1.276	0.041	1.024	0.307					
	Living alone	0.036 (−2.708, 2.780)	1.393	0.001	0.026	0.979					
	Other	−2.031 (−4.744, 0.681)	1.377	−0.051	−1.475	0.141					
	Social support	0.368 (0.180, 0.556)	0.095	0.183	3.854	<0.001					
	Frequency of reading drug instructions	−1.014 (−2.393, 0.364)	0.700	−0.064	−1.449	0.149					
	Ability to understand drug instructions	2.180 (0.473, 3.887)	0.866	0.119	2.516	0.013					
	Self-efficacy for appropriate medication use	0.642 (0.309, 0.974)	0.169	0.179	3.804	<0.001					
	Medical payment method	−0.551 (−2.599, 1.497)	1.039	−0.021	−0.530	0.596					
	Level of detail of medication education received	−0.072 (−1.224, 1.080)	0.585	−0.004	−0.124	0.902					
	Patient participation in medication decision-making	0.748 (0.551, 0.945)	0.100	0.353	7.479	<0.001					

## 4 Discussion

### 4.1 Medication literacy among middle-aged and older patients with CHD and T2DM needs further improvement

This study found that the medication literacy score of middle-aged and older patients with comorbid CHD and T2DM was 74.05 ± 12.23, slightly lower than previously reported findings, which may be related to factors such as study population composition, regional healthcare environment, disease severity, and complexity of medication regimens (25). These findings suggest that medication literacy in people with chronic disease generally remains insufficient and still requires further improvement. Analysis of mean item scores by dimension showed the highest scores in the calculate dimension and the lowest in the access dimension, with communication also performing relatively poorly. One explanation is that, through repeated long-term exposure to medication-related information such as dosage, frequency, and timing, patients gradually develop relatively stable procedural medication skills. In contrast, information access and communication are higher-order interactive abilities that depend more heavily on patients’ cognitive capacity, initiative, and support from the healthcare environment. Previous research has similarly shown that deficits in information access are particularly pronounced among patients with low medication literacy (26). Most participants in this study were older adults. Age-related physiological and cognitive decline, reduced ability to filter health information, and limited digital literacy may all increase barriers to accessing medication information. In addition, under the influence of traditional physician-centered communication models, patients may be more accustomed to passively receiving instructions than to actively engaging in communication. It is recommended to provide diversified, standardized, and age-friendly medication information resources. In addition, standardized medication communication checklists may help strengthen patients’ ability to access and communicate medication-related information in healthcare settings (27).

### 4.2 Factors associated with medication literacy among middle-aged and older patients with CHD and T2DM

#### 4.2.1 Individual factor

##### 4.2.1.1 Educational level

This study found that educational level was significantly associated with medication literacy among middle-aged and older patients with comorbid CHD and T2DM (*β* = 0.218, *p* < 0.05), which is consistent with previous studies (28, 29). A possible explanation is that patients with higher educational attainment tend to have better reading comprehension and analytical skills, which confer advantages in understanding medical advice and medication information, calculating doses, weighing benefits and risks, and identifying and managing adverse reactions. In addition, these patients may place greater importance on health management and are more likely to actively seek individualized medication information through various channels and communicate effectively with medical professionals (30, 31). By contrast, patients with lower educational levels may rely more heavily on support from healthcare professionals or family members. Therefore, in clinical practice, close attention should be paid to the medication education needs of patients with low educational levels. Popularized, visualized, and repeatedly reinforced education methods, such as graphic manuals, short videos, scenario-based demonstrations, and the “teach-back” method, may help to facilitate the effective transmission and mastery of core medication knowledge. Perhaps guiding family members to participate appropriately in patient medication management, strengthening pharmaceutical services in primary healthcare institutions, and developing simple and practical medication assistance tools can help improve medication literacy in patients with low educational levels.

#### 4.2.2 Social factors

##### 4.2.2.1 Social support

This study showed that middle-aged and older patients with comorbid CHD and T2DM have moderate levels of social support, and social support was significantly associated with their medication literacy (*β* = 0.183, *p* < 0.05). This is consistent with previous studies (32, 33). Higher levels of social support may improve medication literacy through objective support, subjective support, and support utilization. Objective support provides medication guidance, supervision, and reminders, helping patients overcome barriers to accessing and using medication information. Subjective support enhances patients’ sense of belonging and self-management efficacy, alleviates negative emotions caused by long-term disease management, and increases willingness to learn medication knowledge and practice standardized drug use. High support utilization enables patients to actively seek help from family, healthcare providers, and community resources, translating external support into practical medication management capabilities (32, 34, 35). Therefore, clinical practice may fully integrate support from family, peers, communities, and healthcare teams, and build a comprehensive social support system through family-based interventions, community pharmaceutical services, post-discharge follow-up, and peer support programs.

##### 4.2.2.2 Place of residence

The results of this study showed an urban–rural binary difference in medication literacy among middle-aged and older patients with comorbid CHD and T2DM, with rural patients having a significantly lower level of medication literacy than urban patients (*β* = −0.126, *p* < 0.05). This finding is consistent with previous studies (36). One possible explanation is that pharmaceutical services in rural areas are relatively underdeveloped, and access to and coverage of health education resources are much lower than in urban areas, making it difficult for patients to receive continuous, systematic, and professional medication guidance (37, 38). Previous studies have shown clear urban–rural disparities in the utilization of health services, quality of chronic disease management, and access to digital health resources. Such structural inequalities may further affect patients’ health literacy and health outcomes (39). Therefore, rural patients should be considered a priority population for interventions, with sustained efforts to strengthen pharmaceutical services in primary healthcare institutions and provide more accessible and age-friendly health education.

#### 4.2.3 Cognitive and functional factors

##### 4.2.3.1 Self-efficacy for appropriate medication use

This study found that self-efficacy for appropriate medication use was significantly associated with medication literacy among middle-aged and older patients with comorbid CHD and T2DM (*β* = 0.179, *p <* 0.05), which is consistent with previous studies (40). Patients with higher self-efficacy for appropriate medication use may be more confident in managing complex medication regimens and more willing to learn medication-related knowledge and follow appropriate medication practices (41). In contrast, patients with lower self-efficacy may be more likely to avoid, hesitate, or rely on others when facing multimorbidity, long-term medication use, and treatment adjustments. Previous studies have also shown that patients with stronger self-efficacy for appropriate medication use are more likely to exhibit a high-medication-literacy profile characterized by active communication, whereas those with lower self-efficacy tend to belong to a low-medication-literacy group marked by limited information access (26). Therefore, clinical practice should emphasize both the assessment and enhancement of medication self-efficacy. Approaches such as motivational interviewing, individualized stepwise management goals, staged positive feedback to accumulate successful experiences, and medication skills training may help patients gradually build confidence and competence in medication management.

##### 4.2.3.2 Ability to understand drug instructions

This study showed that the ability to understand drug instructions was significantly associated with medication literacy among middle-aged and older patients with CHD and T2DM (*β* = 0.119, *p <* 0.05), which is consistent with previous studies (42, 43). Drug instructions are the most direct and commonly used source of medication information for patients outside of the hospital setting. Because patients with CHD and T2DM often use multiple medications over long periods, they have greater need for accurate understanding of medication information. Patients who can better interpret dosage instructions, precautions, contraindications, and potential adverse reactions are more likely to apply medication information correctly in daily life. Most participants in this study were older adults who may have impaired vision or declining cognitive function. Moreover, the existing drug instructions, due to their small font size, dense professional terms, and lack of prominent core information, further exacerbate the negative impact of insufficient understanding ability on medication literacy (42). Therefore, the content presentation and layout design of drug instructions should be optimized in the direction of patient-friendly and older friendly, such as highlighting key warning information, simplifying professional terms, optimizing visual levels, and supporting voice or video explanation QR code, so as to alleviate the reading and understanding barriers of older patients.

#### 4.2.4 Healthcare system factor

##### 4.2.4.1 Degree of patient participation in medication decision-making

This study found that greater patient participation in medication decision-making was associated with better medication literacy (*β* = 0.353, *p* < 0.05). Muscat et al. (44) also pointed out that health literacy and shared decision-making are intrinsically linked. Gentizon et al. (45) argued that the interactive skills underlying medication literacy require patients to actively communicate with healthcare providers, express treatment concerns, and participate in decision-making. Participation in medication decision-making may help patients obtain and understand core medication information and enhance their acceptance of treatment plans (46). Moreover, treatment decisions often need to balance factors such as patients’ financial circumstances, lifestyle, risk preferences, and long-term goals (47). If patient participation is insufficient, their real needs and implementation difficulties may not be incorporated into the decision-making process, thereby reducing the appropriateness and feasibility of the treatment plan. However, despite the broad international advocacy for shared decision-making, its implementation in real-world clinical settings still faces multiple barriers. Previous research has shown that patient participation in medication decision-making among people with chronic diseases remains limited, largely because healthcare providers dominate the decision-making process (48). Whereas, healthcare professionals seldom actively ask about patients’ medication preferences or discuss potential drug side effects, while patients themselves rarely raise medication-related questions or express difficulties in medication use (49). A prior study has confirmed that shared decision-making tools designed to promote patient participation in medication decisions can significantly improve medication literacy (50). Therefore, clinical practice should move away from the paternalistic communication models and actively promote shared decision-making in pharmacotherapy. Decision aids may also be used to enhance patients’ willingness and ability to participate. Such efforts may facilitate the progression of medication literacy from basic functional literacy toward higher levels of interactive and critical literacy.

## 5 Limitations

This study has several limitations. First, the cross-sectional designs can only reveal the correlations between variables, but cannot infer causal relationships. Therefore, the directionality of the relationships observed in this study is not yet clear. Further longitudinal and intervention studies are needed in the future to clarify causal pathways. Second, this was a single-center study using convenience sampling, and all participants were inpatients from one tertiary Grade A hospital, which may have introduced selection bias and limited the representativeness and external validity of the findings. Inpatients in tertiary hospitals may differ from patients in communities or primary healthcare institutions in terms of disease severity, socioeconomic status, and access to medical resources. Therefore, the generalizability of the findings is limited. In the future, multi-center and large sample studies should be carried out in different levels of medical institutions and community populations. Third, some of the assessment tools used in this study were developed in the context of the local healthcare system and cultural setting. Their applicability and comparability may therefore be limited across different countries, healthcare systems, and linguistic or cultural environments. Accordingly, caution should be exercised when comparing the present findings with those of international studies. Fourth, the data were mainly based on patient self-report and may have been affected by social desirability bias and recall bias. Future studies could incorporate objective data to improve the accuracy and reliability of the findings. Fifth, the inclusion of potential associated factors at different levels was not comprehensive, and the complex interrelationships among these factors were not fully explored. Future studies should include more relevant variables to further clarify the mechanisms underlying medication literacy.

## 6 Conclusion

Middle-aged and older patients with comorbid CHD and T2DM have substantial room for improvement in medication literacy, particularly in medication information access and communication. Medication literacy was significantly associated with multilevel factors, including educational level at the individual level; social support and place of residence at the social level; self-efficacy for appropriate medication use and the ability to understand drug instructions at the cognitive and functional level; and patient participation in medication decision-making at the healthcare system level. In clinical and community chronic disease management, medication literacy assessment should be incorporated into the full-cycle management process for middle-aged and older patients with comorbid CHD and T2DM, and individualized, systematic intervention measures should be implemented to improve the level of public medication literacy, promote the safety of home medication, and improve the quality of life.

## Data Availability

The original contributions presented in the study are included in the article/supplementary material, further inquiries can be directed to the corresponding author/s.
